# Secular Trends in Neuroblastoma Mortality Before and After the Cessation of National Mass Screening in Japan

**DOI:** 10.2188/jea.JE20090037

**Published:** 2009-09-05

**Authors:** Kota Katanoda, Kunihiko Hayashi, Keiko Yamamoto, Tomotaka Sobue

**Affiliations:** 1 Cancer Information Services and Surveillance Division, Center for Cancer Control and Information Services, National Cancer Center, Tokyo, Japan; 2 Department of Basic Allied Medicine, School of Health Sciences, Gunma University, Maebashi, Japan

**Keywords:** mass screening, mortality, neuroblastoma

## Abstract

**Background:**

In 2003, the Japanese government halted the national mass screening program for neuroblastoma (NB), which had been running since the mid-1980s. It is not known whether the NB mortality rate subsequently increased or decreased.

**Methods:**

Utilizing vital statistics data from 1980 through 2006, we analyzed the secular trends in NB mortality by using cancer of the adrenal gland as a surrogate. We examined the validity of this substitution by comparing the results with data from death certificates. Using a joinpoint regression model, we examined the trends in age-specific mortality rates by calendar year and cumulative mortality rates by birth year. The cumulative mortality rate was analyzed for age under 1 or 2 years for infants born after the cessation of the mass screening program.

**Results:**

The number of deaths from cancer of the adrenal gland was closely correlated with the number of deaths from NB. Significant decreases in the mortality rate were observed from 1980 through 2006 by calendar year for those aged under 1 year, 1 to 4 years, and 5 to 9 years. The cumulative mortality rates by birth year also significantly decreased from the 1980 birth cohort. Although the cumulative mortality rates under the age of 2 appear to have increased after the 2003 birth cohort, the change was not statistically significant.

**Conclusions:**

No significant increase in the NB mortality rate was detected after the cessation of the mass screening program in Japan. However, continuous monitoring is still needed to fully evaluate this health policy decision.

## INTRODUCTION

Studies conducted in Germany and in the province of Quebec, Canada showed that screening infants for neuroblastoma (NB) did not result in lower NB mortality.^1^^,^^2^ Although a large number of epidemiological studies have been conducted in Japan, the findings regarding the effectiveness of NB screening have been inconsistent.^3^^–^^8^ Clinical studies have reported that a considerable fraction of NB patients whose disease was detected by mass screening had favorable outcomes, which suggests the possibility of over-diagnosis.^9^^–^^14^ In 2003, the Japanese government halted the national mass screening program—which had been in place since the mid-1980s for infants aged 6 months—because of the potential for over-diagnosis and the lack of evidence for its effectiveness in reducing NB mortality.^15^ Most local municipalities in Japan stopped the program during the following year. It is not known whether the NB mortality rate increased or decreased after this national change. Therefore, we analyzed secular trends in NB mortality in Japan before and after the cessation of the national mass screening program.

## METHODS

As is the case in most countries, the Japanese government collects vital statistics data, in which the causes of death are classified according to the International Classification of Diseases (ICD). NB mortality cannot be directly identified in this classification because deaths attributable to NB are coded based on the organ affected, and are grouped together with deaths due to other cancers affecting the same organ.^16^ Two different methods have been adopted to address this issue. The first approach (hereafter referred to as method-1) is to extract the data on deaths due to cancer of the candidate sites, inspect the relevant individual death certificates, and identify NB deaths based on the description of the cause of death or the histological type.^16^ The second approach (hereafter referred to as method-2) is to use deaths from cancer of the adrenal gland as a surrogate index.^17^

Method-2 is less accurate than method-1 because NB can occur at sites other than the adrenal gland, and because other histological types of cancer can occur in this organ. Method-1 requires official permission for the use of unpublished vital statistics data, whereas method-2 uses only published vital statistics data, at least for the years after the ICD version 10 was applied.

The present study used method-1 to analyze data from the year 2006. We obtained individual mortality data from the vital statistics, with official permission, according to the following criteria: year of death = 2006; age at death = 0 to 14 years; cause of death (ICD) = malignant neoplasms of mediastinum (ICD-9 164.2, 164.3, 164.9; ICD-10 C38.1, C38.2, C38.8), connective and soft tissue (ICD-9 171.0, 171.2, 171.3, 171.4, 171.5, 171.6, 171.7, 171.8, 171.9), peripheral nerves and autonomic nervous system (ICD-10 C47.0, C47.1, C47.2, C47.3, C47.4, C47.5, C47.6, C47.8, C47.9), retroperitoneum and peritoneum (ICD-9 158.0, 158.8, 158.9; ICD-10 C48.0, C48.1, C48.2, C48.8), adrenal gland (ICD-9 194.0; ICD-10 C74.0, C74.1, C74.9), or other/ill-defined sites (ICD-9 195.0, 195.1, 195.2, 195.3, 195.4, 195.5, 195.8; ICD-10 C76.0, C76.1, C76.2, C76.3, C76.4, C76.5, C76.7, C76.8). Then, we inspected individual death certificates for the extracted data, and identified NB deaths based on the recorded causes of death or histological types. This process was performed by one of the authors (K. K.) and was confirmed by another author (K. Y., a pediatrician). For the data from 1980 through 2001, we obtained data on NB deaths from previous reports^16^^,^^18^ that had used a method identical to the present method-1 for extracting and identifying NB deaths. Thus, in the present study, data on the number of NB deaths based on method-1 were available for the years from 1980 through 2001, and for the year 2006. We could not apply method-1 to the time period from 2002 through 2005 because a previous application to use death certificates for research had been rejected,^19^ and the document storage period had expired by the time of our application.

For method-2, we calculated the age-specific number of deaths from adrenal gland cancer, based on officially obtained individual mortality data from the vital statistics. The criteria for data collection were as follows: year of death = 1980 to 2006; age at death = 0 to 14 years; cause of death (ICD) = malignant neoplasms of the adrenal gland (ICD-9 194.0; ICD-10 C74.0, C74.1, C74.9).

To validate method-2, we calculated the Pearson correlation coefficient between the number of NB deaths and the number of adrenal gland cancer deaths, using data from 1980 through 2001, and from 2006.

We obtained population data from the published vital statistics and calculated the age-specific mortality rate by calendar year for cancer of the adrenal gland. For the age-specific mortality rate, age was stratified into the following 4 groups: 0 years, 1 to 4 years, 5 to 9 years, and 10 to 14 years. We also calculated the cumulative mortality rate by birth year, by summing the 1-year age-specific mortality rate according to each birth year.^20^ The number of deaths according to each age and each birth year was used as the numerator for the 1-year age-specific mortality rate. The denominator was the number of births (for age younger than 1 year) or the population for each age (for 1 year or older). The most recent birth year that we analyzed was 2005 for the cumulative mortality rate under 1 year of age, 2004 for 2 years of age, 2003 for 3 years of age, and 2002 for 4 years of age.

For the statistical analysis, we used a joinpoint regression model,^21^ implemented in the Joinpoint Regression Program (version 3.3.1) developed by the US National Cancer Institute. This method describes changes in data trends by connecting several different line segments on a log scale at joinpoints. The analysis starts with the minimum number of joinpoints (that is, 0, representing a straight line) and tests for the model fit with a maximum number of joinpoints. A Monte Carlo permutation method is used for tests of significance. In addition, the annual percent change (APC) for each line segment and the corresponding 95% confidence interval (CI) were estimated. In the statistical analysis, the number of deaths was assumed to follow a Poisson distribution. The maximum number of joinpoints was set at 3, the minimum number of observations from a joinpoint to either end of the data was set at 2 (including the end and joinpoint), and the minimum number of observations between 2 joinpoints was set at 4 (including the joinpoints).

## RESULTS

Table 1 shows the secular trends in the age-specific number of deaths, according to the 2 methods. Although method-1 tended to yield slightly larger numbers than method-2, the secular trends were similar. The Pearson correlation coefficients between the annual numbers of deaths according to the 2 methods were close to 1 (1.00 for 0–14 years, 0.96 for <1 year, 0.98 for 1–4 years, 0.96 for 5–9 years, and 0.73 for 10–14 years; all 5 correlation coefficients were significantly different from 0 [number of data points = 23, *P* < 0.001]). In 2006, 45 deaths from cancer of the adrenal gland were observed, one of which was non-NB (malignant pheochromocytoma). In comparison, 45 NB deaths were identified by the inspection of death certificates from 2006, of which 1 case occurred at a site other than the adrenal gland (retroperitoneum).

**Table 1.** Numbers of deaths due to neuroblastoma (NB) and adrenal gland cancer from 1980 to 2006

Calendar year^a^	1) Number of deaths from NB identified by individual death certificates^b^					Calendar year^a^	2) Number of deaths from cancer of the adrenal gland^c^				
	
	0–14 years	<1 year	1–4 years	5–9 years	10–14 years		0–14 years	<1 year	1–4 years	5–9 years	10–14 years
1980–1982	381	28	209	119	25	1980–1982	338	22	194	103	19
1983–1985	329	31	174	102	22	1983–1985	294	28	150	94	22
1986–1988	280	15	132	101	32	1986–1988	249	15	119	88	27
1989–1991	221	10	109	81	21	1989–1991	207	8	106	74	19
1992–1994	178	11	72	77	18	1992–1994	171	8	68	76	19
1995–1997	185	16	67	78	24	1995–1997	181	14	65	79	23
1998–2000	147	9	55	59	24	1998–2000	139	8	53	56	22
2001–2003	(N.A.)	(N.A.)	(N.A.)	(N.A.)	(N.A.)	2001–2003	118	6	49	42	21
2004–2006	(N.A.)	(N.A.)	(N.A.)	(N.A.)	(N.A.)	2004–2006	123	7	55	45	16
2001, 2006	86	4	38	29	15	2001, 2006	84	4	38	27	15

^a^Consecutive 3-year periods from 1980 to 2006 were pooled. Two years (2001 and 2006) were also pooled.

^b^The numbers of NB deaths between 1980 and 2001 were obtained from previous reports (Hanawa et al, 1990 and Hayashi et al, 2004), and the numbers of NB deaths in 2006 were counted by one of the authors (K. K.).

^c^Deaths due to cancer of the adrenal gland were defined by codes in the ICD: 194.0 for 1980–1994, C74.0, C74.1, and C74.9 for 1995–2006.

N.A.: Not available

**Figure 1.**
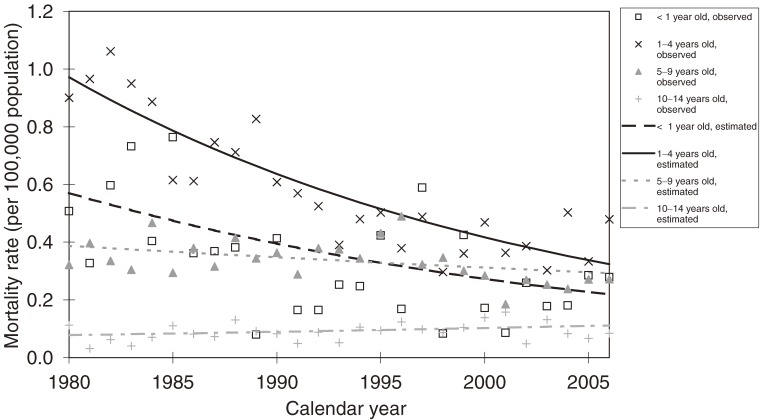
Annual trends in age-specific mortality rate for cancer of the adrenal gland, by calendar year

**Figure 2.**
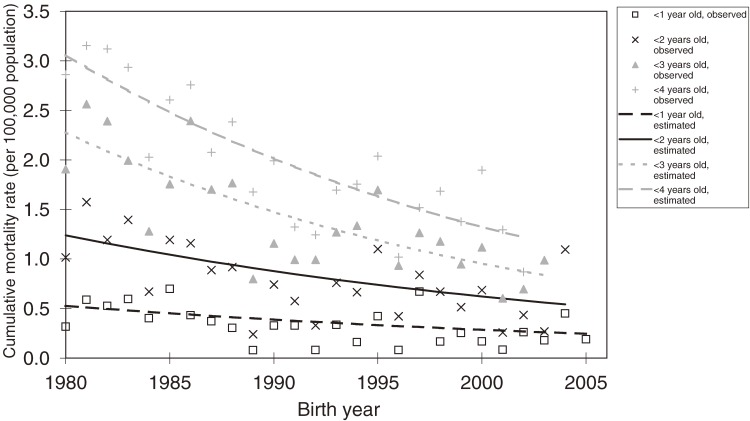
Annual trends in cumulative mortality rate for cancer of the adrenal gland, by birth year. Note: The most recent analyzed birth year was 2005 for age <1 year, 2004 for age <2 years, 2003 for age <3 years, and 2002 for age <4 years

Table 2 shows the results of the joinpoint analysis. Significant decreases in the age-specific mortality rate were observed from 1980 through 2006 by calendar year for those aged less than 1 year, 1 to 4 years, and 5 to 9 years. The cumulative mortality rates by birth year also significantly decreased from the 1980 birth cohort. Figure 1 shows the trends in the age-specific mortality rate. The mortality rates for 1 to 4 years of age in 2004 and 2006 were high, but no significant increase or joinpoint was detected around this time. Figure 2 shows the trends in the cumulative mortality rate. The cumulative mortality rate under the age of 2 for the 2004 birth cohort was considerably higher than the cumulative mortality rate for the past several birth cohorts. However, this change was not detected as a significant joinpoint.

**Table 2.** Results of joinpoint regression analysis for the secular trends in adrenal gland cancer mortality

A) Age-specific mortality rate, by calendar year						

	Number of joinpoints	Line segment		Annual % change	95% confidence interval	
		
		Start	End		Lower	Upper
0 year old	0	1980	2006	−3.6^a^	−5.8	−1.4
1–4 years old	0	1980	2006	−4.1^a^	−5.0	−3.3
5–9 years old	0	1980	2006	−1.1^a^	−2.0	−0.1
10–14 years old	0	1980	2006	1.4	−0.4	3.2


B) Cumulative mortality rate, by birth year^b^						

	Number of joinpoints	Line segment		Annual % change	95% confidence interval	
		
		Start	End		Lower	Upper

<1 year old	0	1980	2005	−3.0^a^	−5.4	−0.5
<2 years old	0	1980	2004	−3.4^a^	−5.3	−1.4
<3 years old	0	1980	2003	−4.3^a^	−5.7	−2.8
<4 years old	0	1980	2002	−4.1^a^	−5.2	−2.9

^a^Annual % change is statistically significant from zero.

^b^The analyzed most recent birth year was 2005 for age <1 year, 2004 for <2 years, 2003 for age <3 years, and 2002 for age <4 years.

## DISCUSSION

We examined the secular trends in the mortality rate for NB, using mortality for cancer of the adrenal gland as a surrogate index, and found no significant increase before or after the cessation of the Japanese national mass screening program. We confirmed the validity of this surrogate method by examining the correlation between the numbers of deaths from the 2 cancers. Nationwide mass screening had previously been performed for infants aged 6 months, and the participation level was high (ranging from 84% to 90% in the period from 1990 through 2001). Because the most recent year of death that we analyzed was 2006, any increase in the age-specific mortality rate associated with the cessation of mass screening in 2003 would have been expected to occur among children aged 1 to 4 years. However, we did not observe any significant increase or joinpoint in the mortality rate among this age group around this time. It is possible that the time elapsed since the cessation of mass screening was still too short to detect any increase in mortality for this age group, which included both screened and unscreened individuals, even at the end of our observation period.

The cumulative mortality rate according to birth year was a more direct index to examine the effect of the cessation of the mass screening program. It decreased significantly throughout the observed birth years, and the birth year 2003 was not detected as a significant joinpoint. However, these results should be interpreted with caution because the analyzed range of birth years and ages was limited after the cessation of the mass screening program. In consideration of the time from diagnosis to death, we should assume that any increase in the mortality rate for infants born after the cessation could occur later than the end of our observation period.

There are several Japanese municipalities that continued mass screening for NB after 2003. However, the number of such municipalities is very small, and our results did not change when we excluded the prefectures to which these municipalities belong (ie, Hokkaido, Kanagawa, Niigata, Shizuoka, Kyoto, Osaka, and Kumamoto).

We found a high correlation between the number of deaths from NB and the number of deaths from adrenal gland cancer, with the former slightly and consistently higher than the latter (Table 1). This tendency was in agreement with a previous report, in which approximately 90% of NB deaths were attributable to cancer of the adrenal gland, and almost all of the cancers that occurred at this site were NB.^16^ One death due to non-NB cancer in the adrenal gland was included among the data for 2006, but this was considered to be an unusual case.

The effect of the cessation of the mass screening program for NB should be verified by monitoring the trend in incidence.^22^ Studies have reported the trends in NB incidence and mortality before and after the start of a national mass screening program, based on data from a population-based cancer registry.^3^^,^^5^ Additional similar studies need to be conducted, and should include a sufficient observation period after the cessation of the program.

In conclusion, no significant increase in the NB mortality rate was detected after the cessation of the national mass screening program in Japan. However, continuous monitoring is still needed to evaluate further this health policy decision.
